# Environmental Enrichment Attenuates Oxidative Stress and Alters Detoxifying Enzymes in an A53T α-Synuclein Transgenic Mouse Model of Parkinson’s Disease

**DOI:** 10.3390/antiox9100928

**Published:** 2020-09-28

**Authors:** Jung Hwa Seo, Seong-Woong Kang, Kyungri Kim, Soohyun Wi, Jang Woo Lee, Sung-Rae Cho

**Affiliations:** 1 Department and Research Institute of Rehabilitation Medicine, Yonsei University College of Medicine, Seoul 03722, Korea; zugula@yuhs.ac (J.H.S.); KSWOONG@yuhs.ac (S.-W.K.); kby930@yuhs.ac (K.K.); wish118@yonsei.ac.kr (S.W.); 2 Brain Korea 21 PLUS project for Medical Science, Yonsei University College of Medicine, Seoul 03722, Korea; 3 Department of Rehabilitation Medicine, Gangnam Severance Hospital, Yonsei University College of Medicine, Seoul 06273, Korea; 4 Rehabilitation Institute of Neuromuscular Disease, Yonsei University College of Medicine, Seoul 03722, Korea; 5 Department of Medicine, The Graduate School, Yonsei University, Seoul 03722, Korea; 6 Department of Physical Medicine and Rehabilitation, National Health Insurance Service Ilsan Hospital, Goyang 10444, Korea; 7 Graduate Program of Nano Science and Technology, Yonsei University, Seoul 03722, Korea

**Keywords:** Parkinson’s disease, environmental enrichment, oxidative stress, detoxifying enzymes

## Abstract

Although environmental enrichment (EE) is known to reduce oxidative stress in Parkinson’s disease (PD), the metabolic alternations for detoxifying endogenous and xenobiotic compounds according to various brain regions are not fully elucidated yet. This study aimed to further understand the role of EE on detoxifying enzymes, especially those participating in phase I of metabolism, by investigating the levels of enzymes in various brain regions such as the olfactory bulb, brain stem, frontal cortex, and striatum. Eight-month-old transgenic PD mice with the overexpression of human A53T α-synuclein and wild-type mice were randomly allocated to either standard cage condition or EE for 2 months. At 10 months of age, the expression of detoxifying enzymes was evaluated and compared with wild-type of the same age raised in standard cages. EE improved neurobehavioral outcomes such as olfactory and motor function in PD mice. EE-treated mice showed that oxidative stress was attenuated in the olfactory bulb, brain stem, and frontal cortex. EE also reduced apoptosis and induced cell proliferation in the subventricular zone of PD mice. The overexpression of detoxifying enzymes was observed in the olfactory bulb and brain stem of PD mice, which was ameliorated by EE. These findings were not apparent in the other experimental regions. These results suggest the stage of PD pathogenesis may differ according to brain region, and that EE has a protective effect on the PD pathogenesis by decreasing oxidative stress.

## 1. Introduction

Parkinson’s disease (PD) is the second most common neurodegenerative disease after Alzheimer’s disease, affecting about 1–2% of the population older than 60 years of age [1,2]. PD is considered an intrusive condition that involves multiple functional systems. It is characterized by four cardinal motor symptoms: resting tremor, bradykinesia, rigidity, and postural instability [3]. Recently, diverse non-motor symptoms, such as autonomic dysfunction, gastrointestinal symptoms, cognitive dysfunction, sleep disturbance, and neuropsychiatric symptoms have been proposed as accompanying presentations in PD [4].

Despite a wide range of studies on the pathophysiology of PD, the exact mechanisms of PD have not yet been elucidated. Various hypotheses have been suggested, including altered metabolism [5]. Because various endogenous and environmental toxic materials are responsible for neuronal degeneration, detoxification is believed to represent an important defensive mechanism against PD [6,7]. Detoxifying metabolism is largely divided into three phases: phase I modifies toxins into active metabolites via hydrolysis, oxidation, and reduction; phase II catalyzes the products derived from phase I into hydrophilic products with transferase enzymes; and phase III excretes the final products from cells via various transporter systems [8].

Classically, it is thought that PD is caused by the loss of dopaminergic neurons in the substantia nigra pars compacta, with typical motor symptoms induced by disruptions of the basal ganglia. In more recent research studies, symptoms related to olfactory structures and the gastrointestinal tract have been proposed as induction sites of PD [9,10,11]. The olfactory bulb acts as an entry site for external materials, delivering signals from the olfactory mucosa, which is directly exposed to the external environment, into the olfactory cortex [12]. In addition, it provides protection against harmful substances by secreting antioxidant and detoxifying enzymes [13]. The detoxifying properties of the olfactory bulb play an important role against various neurodegenerative diseases associated with xenobiotics and environmental toxins [14]. Non-motor symptoms of PD, such as hyposmia, a representative non-motor symptom of PD that develops in the premotor period, can precede the development of typical motor symptoms by up to several decades [15].

Environmental enrichment (EE), a combination of physical, cognitive, and social interactions, is generally accepted as a typical model of rehabilitation in animal models [16]. Functional improvements in neurological diseases have been proven by EE in several studies [17]. Moreover, various neurochemical and histological hypotheses have been suggested for the underlying mechanisms of this improvement, including reduced oxidative stress, anti-neuroinflammation, neurogenesis, synaptic plasticity, and neuroprotection via the induction of neurotrophic factors [18,19,20,21].

In a preliminary study investigating the olfactory bulb of normal adult mice, exposure to EE for 2 months resulted in the significant up-regulation of several detoxifying enzymes of phase I and phase II compared with the wild-type control group [13]. In another study using transgenic PD mice that overexpress human A53T α-synuclein, however, several detoxifying enzymes were found to be significantly increased in the olfactory bulb of PD mice compared to wild-type mice at the early stages of the disease, which were less enhanced in the later stages. EE was found to offset this up-regulation, resulting in the normalized levels of detoxifying enzymes [22].

This study aims to expand on preliminary findings of the activities of detoxifying enzymes in various dopaminergic structures, including the brain stem, frontal cortex, and striatum, in addition to the olfactory bulb. In particular, we focused on the phase I metabolism, an initial stage of detoxification. The effect of EE on enzymatic activity, oxidative stress, neurogenesis, neuroprotection, and functional ability were also investigated to determine their significance in PD rehabilitation.

## 2. Materials and Methods

### 2.1. Transgenic Mouse Model of Parkinson’s Disease

To generate wild-type and transgenic mice, we used the human α-synuclein A53T transgenic line G2-3 (B6.Cg-Tg [Prnp-SNCA*A53T] 23 Mkle/J; Jackson Laboratories, stock no. 006823, Bar Harbor, ME, USA). The transgenic mice used in this model were heterozygous offspring of an overexpressed copy of the A53T mutant mice. All animals were raised in a facility accredited by the Association for Assessment and Accreditation of Laboratory Animal Care under alternate 12-h light and dark cycles, with free access to food and water. The experimental procedures were approved by the Institutional Animal Care and Use Committee (IACUC 2017-0039).

### 2.2. Mice Rearing and Sacrifice

**Figure 1:**
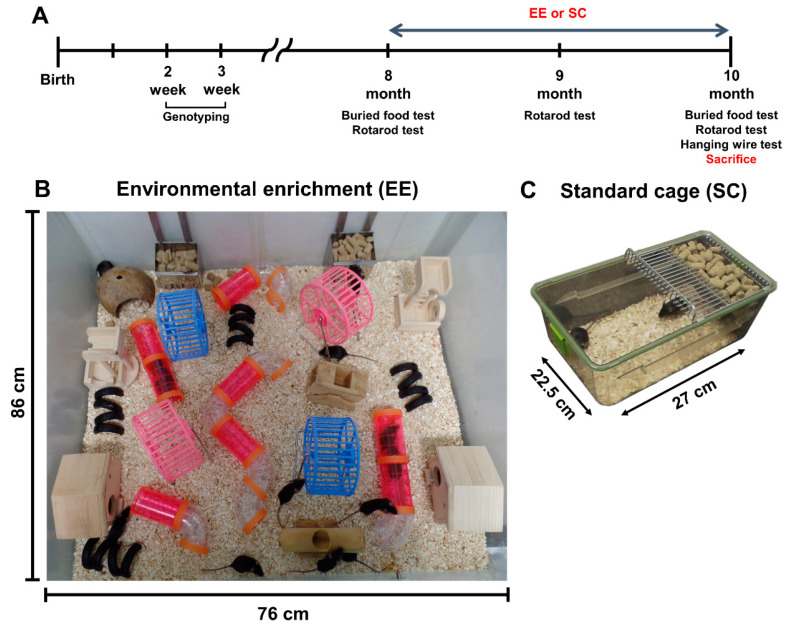
Experimental design. (**A**) Schematic timeline of the experiment in a mouse model of Parkinson’s disease (PD). All subjects were sacrificed at the age of 10 months after different housing for 2 months. (**B**) Environmental enrichment (EE; 86 × 76 × 31 cm^3^) including shelters, tunnels, toys, running wheels for voluntary exercise, and social interaction. (**C**) Standard cage (SC; 27 × 22.5 × 14 cm^3^).

EE was established by placing the mice in large cages (86 × 76 × 31 cm^3^) with various equipment, such as tunnels, shelters, toys, and running wheels [16]. These objects were provided for voluntary exercise and social interaction. Only 12 to 15 mice were reared in each EE cage. In contrast, the control mice were housed in 27 × 22.5 × 14 cm^3^ standard cages (SC) without social interaction (3 to 5 mice per cage). All transgenic and wild-type mice were reared in either the EE or SC cages from the age of 8 months to 10 months (Figure 1). The experimental groups were as follows: wild-type mice reared in SC (WT-SC), wild-type mice reared in EE (WT-EE), transgenic mice reared in SC (PD-SC), and transgenic mice reared in EE (PD-EE). The mice were sacrificed at 10 months of age. For molecular study, animals were euthanized and then perfused with 1× phosphate-buffered saline (PBS). The brains were extracted and dissected into four regions including olfactory bulb, brain stem, frontal cortex, and striatum. For analysis of histology, animals were euthanized, perfused with 1× PBS, and re-perfused with 4% paraformaldehyde.

### 2.3. RNA Extraction

The mouse brain sections including olfactory bulb, brain stem, frontal cortex, and striatum were homogenized in Trizol Reagent (Invitrogen Life Technologies, Carlsbad, CA, USA) using sterile homogenizer tips and an electrodynamic cordless motor (cat. no. 749540-0000; Kimble, Vineland, NJ, USA). Extracted total RNA pellets were dissolved in autoclaved DEPC water. The A260:A280 ratio of RNA samples was measured using an Agilent 2100 Bioanalyzer (Agilent Technologies, Palo Alto, CA, USA). The RNA samples were aliquoted and stored at −80 °C until use.

### 2.4. Quantitative Real-Time Polymerase Chain Reaction (qRT-PCR)

Complementary DNA (cDNA) synthesis was performed using Prime Script Reverse Transcriptase (TAKARA). cDNA was subjected to RT-PCR using LightCycler 480 SYBR Green master mix (Roche Applied Science) and Gene Amp PCR system 9700 (Applied Biosystems/Life Technologies, Carlsbad, CA, USA), according to the manufacturer’s instructions. The quantitative expression of the seven enzymes of phase I metabolism related with detoxification, namely, cytochrome P450 family 1 subfamily A member 2 (*Cyp1a2*), carbonyl reductase2 (*Cbr2*), crystallin lambda 1 (*Cryl1*), paraoxonase 1 (*Pon1*), alcohol dehydrogenase 1 (*Adh1*), and aldehyde dehydrogenase 1A7 (*Aldh1a7*), was evaluated by qRT-PCR. The primer sequences used are listed in Table 1. The thermocycler conditions were as follows: denaturation for 5 min at 95 °C, followed by 40 cycles at 95 °C for 5 s, at 62 °C for 20 s, and at 75 °C for 15 s. Melting curve analysis began at 95 °C for 15 s, followed by 1 min at 60 °C. The expression level of each genes was calculated using 2^–△△Ct^ method and normalized relative to the expression of glyceraldehyde-3-phosphate dehydrogenase (*Gapdh*).

### 2.5. Immunohistochemistry (IHC)

After cardiac perfusion, extracted brains were soaked in 6% sucrose for 1 day, followed by soaking in 30% sucrose until sinking completely. The tissues were then frozen in section compound (Leica, Wetzlar, Germany) and cryosectioned at 16 μm thickness along the sagittal or coronal plane using cryomicrotome (Cryostat Leica 1860; Leica Biosystem, Buccinasco, MI, Italy). Immunohistochemistry staining was performed on four sections over a range of > 128 μm.

To evaluate cell proliferation, we stained the sections with Ki-67 (Leica Biosystems, Newcastle, United Kingdom), a cell cycle-associated protein and a neurogenesis marker [23], in the subventricular zone (SVZ), where neurogenesis is known to occur in the adult brain [24]. For the analysis of apoptotic cells, we stained the brain sections using fluorometric terminal deoxynucleotidyl transferase deoxyuridine triphosphate (dUTP) nick end labeling (TUNEL; Promega, Madison, WI, USA) staining, according to the manufacturer’s instructions. Tissue samples were mounted on glass slides with fluorescent mounting medium containing 4′,6-diamidino-2-phenylindole (DAPI; Vectorshield, Vector, Burlingame, CA, USA). The number of Ki-67- and TUNEL-positive cells were counted in the SVZ of brain sections, and the stained sections were analyzed using confocal microscopy (LSM 700, Zeiss).

### 2.6. Glutathione Activity Assay

The ratio of reduced glutathione (GSH) to oxidized glutathione (GSSG) is used as a marker of oxidative stress. We measured total glutathione and GSSG using a glutathione detection kit (ADI-900–160; Enzo Life Science, East Farmingdale, NY, USA) by measuring the absorbance of the reaction products at 405 nm, according to the manufacturer’s instructions. The GSH was obtained by the following formula: GSH = total glutathione − GSSG, in the kit protocols.

### 2.7. Neurobehavioral Tests

#### 2.7.1. Buried Food Test

**Figure 2:**
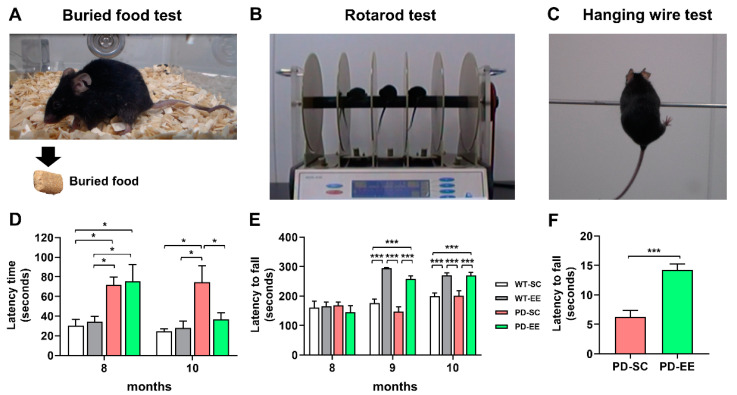
Environmental enrichment (EE) enhanced neurobehavioral functions in PD mice. (**A–C**) Representative pictures of buried food, rotarod, and hanging wire tests. (**A**) Buried food test for the evaluation of olfactory function. (**B**) Rotarod test for the evaluation of motor coordination and balance. (**C**) Hanging wire test for the assessment of the neuromuscular strength in the paws of mice and balance. (**D**) Buried food test result showed the latency time of finding food in PD mice at 8 months of age showed that there were no differences between standard cage (SC) and EE conditions in both wild-type (WT) and PD mice. However, EE for 2 months significantly reduced the latency time in PD-EE mice compared with PD-SC mice (WT-SC, *n* = 6; WT-EE, *n* = 5; PD-SC, *n* = 8; PD-EE, *n* = 8). (**E**) In the rotarod test, at 8 months of age, no significant differences were observed among the groups. However, at 9 and 10 months of age, a better outcome was obtained in the WT-EE and PD-EE mice compared to the WT-SC and PD-SC mice (WT-SC, *n* = 6; WT-EE, *n* = 5; PD-SC, *n* = 6; PD-EE, *n* = 5). * *p* < 0.05; *** *p* < 0.001; one-way ANOVA followed by post-hoc comparison. (**F**) Hanging wire tests evaluated at 10 months of age showed similar results. The PD-EE mice showed improved motor function compared with the PD-SC mice (*n* = 5, each). *** *p* < 0.001; Student’s *t*-test.

The buried food test was performed as described in previous studies [22]. Olfactory function was evaluated using the buried food test, which was used to determine how quickly an overnight-fasted mouse could find a small palatable food that was hidden underneath a layer of bedding (Figure 2A) [25]. The test was performed twice on mice at 8 and 10 months of age. Mice were fasted for 14-18 h the day before testing. The mice were individually placed in a clean holding cages for 5 min, and then transferred to the test cage for 2 min. The pellet was placed in a holding cage and randomly embedded 0.5 cm below the bedding at non-learned positions. We placed the mice in the center of the test cage and recorded the time to find the pellet for 5 min.

#### 2.7.2. Rotarod Test

The rotarod (no. 47600; UGO Basile, Comerio, VA, Italy) test was used to evaluate the motor coordination and balance of the experimental mice using accelerating (4–40 rpm) speed paradigms. After the placing mice on the rotating rods, we measured the time taken for the mice to fall from the rods for 300 s (Figure 2B).

#### 2.7.3. Hanging Wire Test

The hanging wire test evaluated the neuromuscular strength of the paws of the experimental mice. To this end, mice were suspended on a horizontal rod (5 × 5 mm area, 35 cm long, between two 50-cm high poles), and the suspension latencies were measured for 5 min (Figure 2C) [26].

### 2.8. Statistical Evaluation

Statistical analyses were performed using Statistical Package for Social Sciences software version 25.0 (IBM Corporation, Armonk, NY, USA). The variables of behavioral assessments were compared between groups by a Student’s *t*-test. For comparison among three experimental groups in molecular and histological assessments, we used one-way analysis of variance (ANOVA) with least significant difference (LSD) for post-hoc comparison.

## 3. Results

### 3.1. EE-Treated PD Mice Had Improved Neurobehavioral Outcomes

At the age of 8 months and before EE intervention, we performed the neurobehavioral assessment of the experimental mice using buried food and rotarod tests. In the buried food test, there were no baseline differences in the olfactory function between WT-SC mice and WT-EE mice, and between PD-SC mice and PD-EE mice at 8 months, but olfactory dysfunction was shown in PD mice compared to WT mice before exposure to EE. EE treatment for 2 months showed improvement in olfactory function in PD mice (Figure 2D). In the rotarod test, there were no differences among the groups at the age of 8 months; however, motor function was improved by EE in both WT and PD mice at the age of 9 and 10 months (Figure 2E). The hanging wire test showed that EE increased duration time to hang the wire in PD-EE mice compare with PD-SC mice at the age of 10 months (Figure 2F). These results suggested that EE treatment for 2 months improved olfactory and motor functions in PD mice.

### 3.2. EE Attenuated Oxidative Stress in Olfactory Bulb, Brain Stem, and Frontal Cortex but not in the Striatum of PD Mice

**Figure 3:**
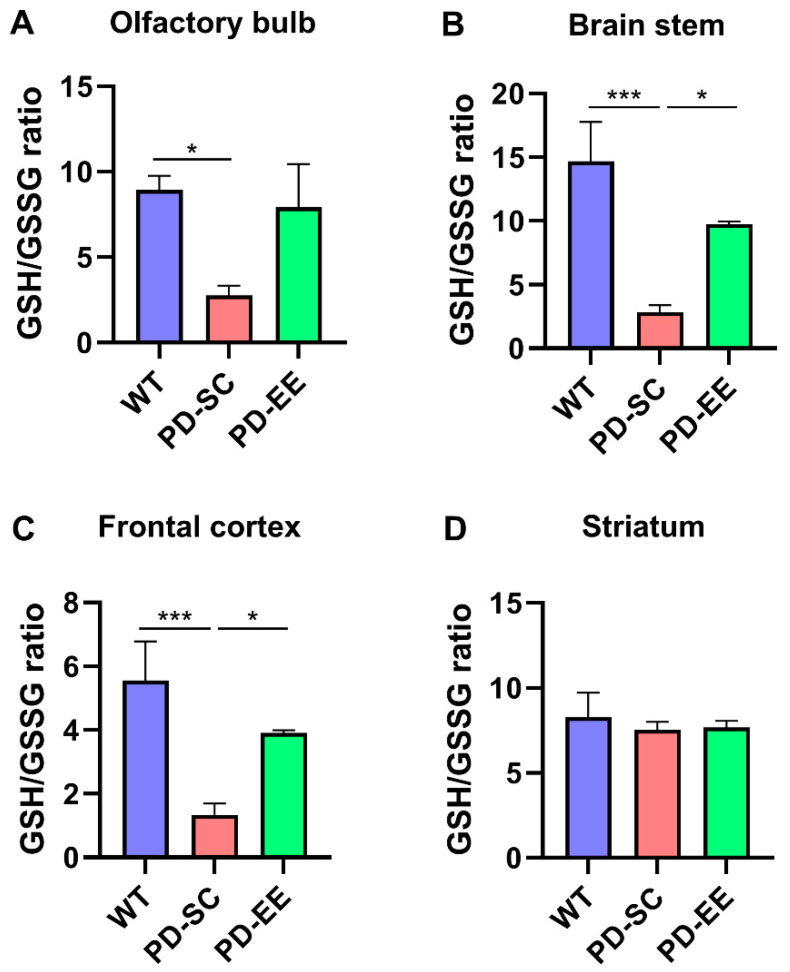
EE attenuated oxidative stress in the brain regions, except for the striatum. (**A**) The glutathione (GSH)/oxidized glutathione (GSSG) ratio was lower in the PD-SC mice than the WT mice in the olfactory bulb. (**B**) GSH/GSSG was lower in the PD-SC compared to the WT and PD-EE in the brain stem. (**C**) GSH/GSSG was lower in the PD-SC compared to the WT and PD-EE in the frontal cortex (**D**) In the striatum, no significant difference in GSH/GSSG ratio was observed among the experimental group (*n* = 3, each). These results suggested that EE attenuated oxidative stress in the olfactory bulb, brain stem, and frontal cortex, but not in the striatum of PD mice. * *p* < 0.05; *** *p* < 0.001; one-way ANOVA followed by post-hoc comparison.

The GSH/GSSG ratio is inversely correlated with oxidative stress and was found to be significantly lower in the PD-SC mice than the WT mice in the olfactory bulb. In the brain stem and frontal cortex, the ratio was lower in the PD-SC mice compared to both WT and PD-EE. However, difference in GSH/GSSG ratio was not observed in the striatum among the groups (Figure 3). These data indicated that EE attenuated oxidative stress in the olfactory bulb, brain stem, and frontal cortex, but not in the striatum of PD mice.

### 3.3. Apototic Cell Death Was Decreased, and Cell Proliferation Was Preserved by EE in the SVZ

**Figure 4:**
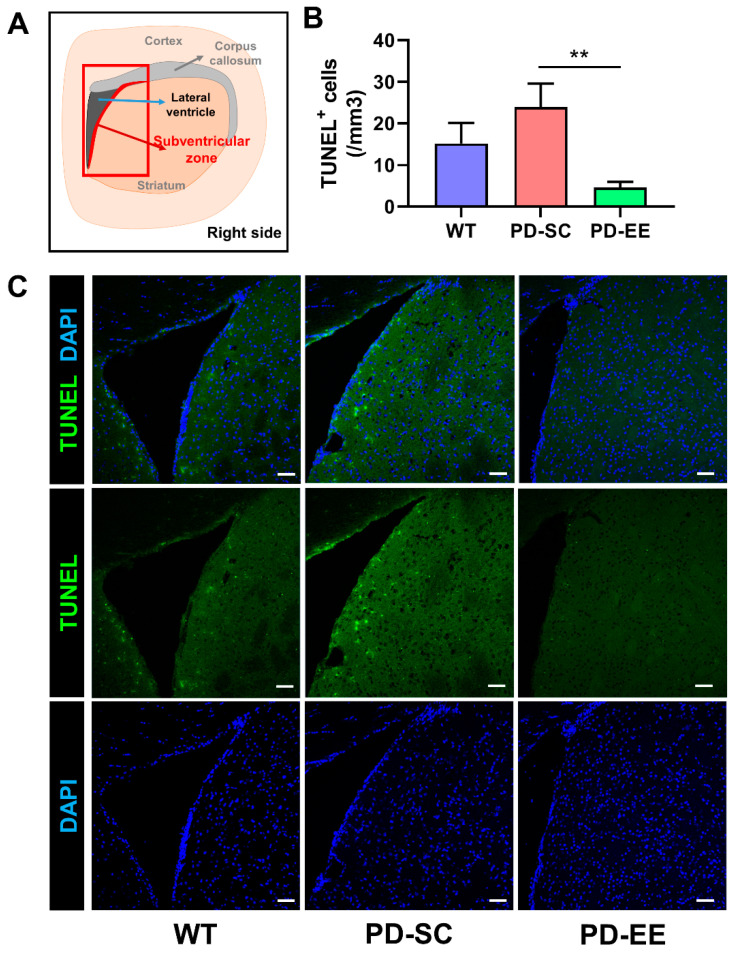
EE decreased apoptotic cell death in the subventricular zone (SVZ) of PD mice. (**A**) The number of terminal deoxynucleotidyl transferase deoxyuridine triphosphate (dUTP) nick end labeling (TUNEL)^+^ cells were evaluated in the SVZ. (**B**) The number of TUNEL^+^ cells were significantly decreased in the PD-EE mice compared to the PD-SC mice (*n* = 3, each). (**C**) Representative confocal microscopic images of TUNEL^+^ cells in the SVZ of coronal sections. Blue color indicates 4′,6-diamidino-2-phenylindole (DAPI)^+^ nuclei. The scale bar represents 50 μm. ** *p* < 0.01; one-way ANOVA followed by post-hoc comparison.

**Figure 5:**
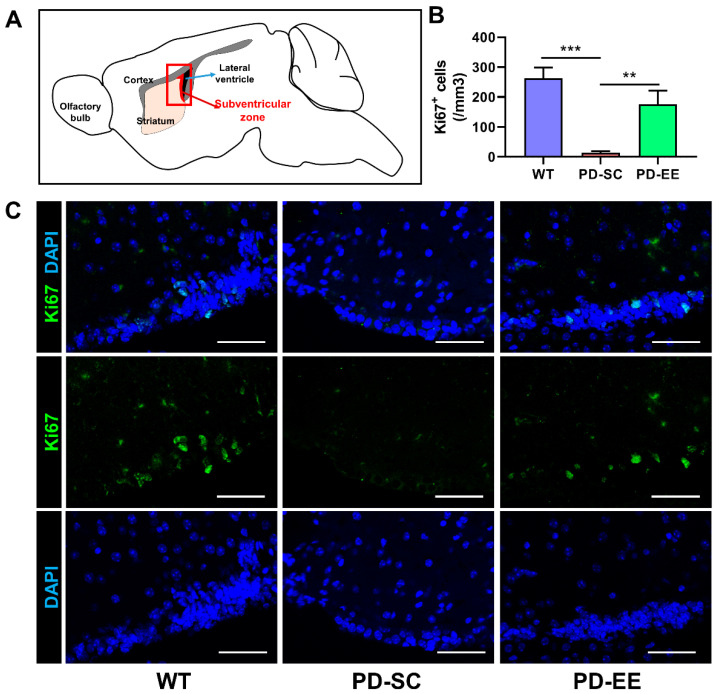
EE preserved cell proliferation in the SVZ of PD mice. (**A**) The density of Ki-67 was evaluated in the SVZ. (**B**) The density of Ki-67 was significantly lower in the PD-SC mice compared with both the WT and PD-EE mice. No significant differences were observed between the WT and PD-EE mice (*n* = 3 each). (**C**) Representative confocal microscopic images of Ki-67^+^ cells in the SVZ of sagittal sections. Astrocytes are green in color. Blue color indicates DAPI^+^ nuclei. The scale bar represents 50 μm. ** *p* < 0.01; *** *p* < 0.001; one-way ANOVA followed by post-hoc comparison.

The concentration of TUNEL-positive cells was significantly lower in the PD-EE mice than the PD-SC mice. No significant differences between the WT and PD-SC mice and between the WT and PD-EE mice were observed (Figure 4). Moreover, the concentration of Ki-67-positive cells was significantly lower in the PD-SC mice. EE was found to ameliorate this deterioration (Figure 5). Taken together, we found that EE had neuroprotective effects through decrease of apoptosis and preservation of cell proliferation in the SVZ of PD mouse brain.

### 3.4. Expression of Detoxifying Enzymes Was Normalized in The Brain of EE-treated PD Mice

**Figure 6:**
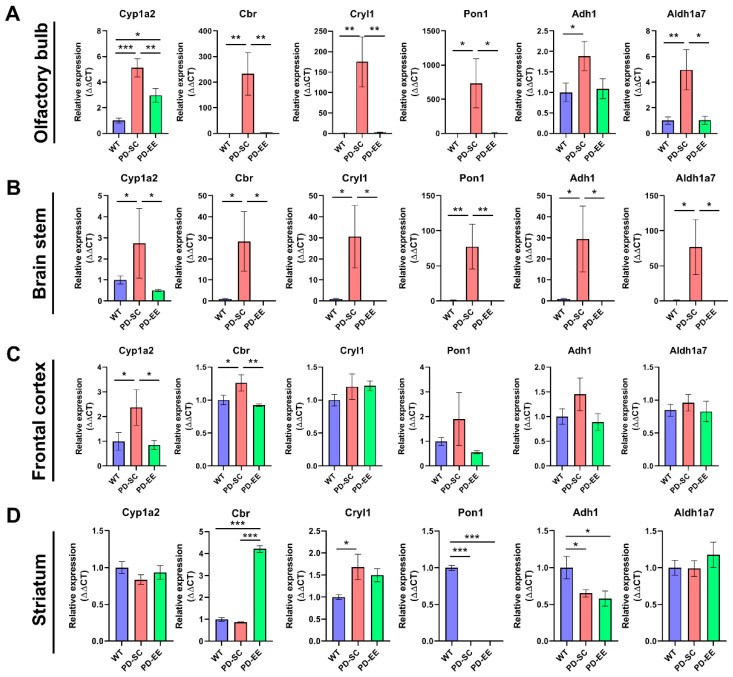
EE altered levels of detoxifying enzymes in the brain tissues of PD mice. (**A**,**B**) In the olfactory bulb and brain stem, we observed significant overexpression of all examined enzymes in the PD-SC, and EE significantly offset these up-regulation, except *Adh1* in the olfactory bulb (*n* = 3–4). (**C**,**D**) These tendencies were not apparent in the frontal cortex and striatum. In the frontal cortex, the levels of only *Cyp1a2* and *Cbr2* were higher in the PD-SC compared both WT and PD-EE. Moreover, in the striatum, several enzymes such as *Pon1* and *Adh1*, were overexpressed in the WT compared to the other groups (*n* = 3–4). * *p* < 0.05, ** *p* < 0.01, *** *p* < 0.001; one-way ANOVA followed by post-hoc comparison. *Cyp1a2*, cytochrome P450 family 1 subfamily A member 2; *Cbr2*, carbonyl reductase2; *Cryl1*, crystallin lambda 1; *Pon1*, paraoxonase 1; *Adh1*, alcohol dehydrogenase 1; *Aldh1a7*, aldehyde dehydrogenase 1A7.

In the olfactory bulb of the PD-SC mice, the expression of the all examined enzymes were up-regulated compared to the WT mice. Except for *Adh1*, the expression of enzymes was significantly lowered by EE. The levels of *Cbr2*, *Cryl1*, and *Aldh1a7* in the PD-EE mice reached to those of the WT; on the other hand, the level of *Cyp1a2* of the PD-EE did not reach to that of the WT. In the brain stem, all examined enzymes were up-regulated in the PD-SC and lowered by EE to the levels of the WT mice. In the frontal cortex, the levels of *Cyp1a2* and *Cbr2* were significantly higher in the PD-SC compared to both WT and PD-EE. The other enzymes were not found to be different expressions among the groups. Lastly, in the striatum of the WT mice, *Pon1* and *Adh1* were expressed more highly than in both PD-SC and PD-EE. The level of *Cryl1* was higher in the PD-SC compared to the WT. Moreover, *Cbr2* was expressed higher in the PD-EE compared to both WT and PD-SC (Figure 6). These results indicated that the expression of detoxifying enzymes associated with phase I of the metabolizing process was altered by EE in the brain regions.

## 4. Discussion

Both clinical and experimental evidence of the impact of physical exercise on PD are increasing, whereby exercise has been found to induce various neuroprotection mechanisms [27,28]. Physical activity not only improves motor function, but also enhances non-motor symptoms and cognitive function in PD [29,30]. Evidence is increasingly recommending the rehabilitation for PD patients as a complementary and indispensable treatment, not merely as an ancillary modality to pharmacological and surgical treatments [31]. Exercise induces neuroplasticity, although the exact mechanism still needs to be clarified. The best-known effect of exercise on brain plasticity is via neurotrophic factors. Various endogenous neurotrophins, such as brain-derived neurotrophic factor, glial cell line-derived neurotrophic factor, insulin-like growth factor-1, and vascular endothelial growth factor are upregulated by exercise [32]. These substances prolong the survival of dopaminergic neurons and increase dopamine levels by stabilizing the intracellular concentration of calcium, reducing oxidative stress, suppressing neuroinflammation, inducing mitochondrial function, and consequently promoting neurogenesis [28]. Moreover, in human studies, increases in the binding potential of dopamine receptors and structural changes via the rehabilitation have been observed in PD patients [33,34,35]. Already, many studies have revealed the effects of EE, a representative rehabilitation model in animal study, on neuroprotection via various mechanisms such as cell proliferation, inhibition of apoptosis, and antioxidation [20,36,37,38,39,40], which were also apparent in our study.

In addition, numerous pieces of evidence have demonstrated that precursor cell proliferation was impaired by the onset of PD [41], and that apoptosis was induced by exposure to toxic compounds in PD [42,43,44,45]. Other previous studies suggested that EE inhibits apoptosis, enhances neuroprotection in the brain of rodent PD models [39,46], and improves neurobehavioral functions in brain disorders including PD [13,47]. It has been suggested that EE can also be the key to provide for the stimulation of various brain regions [13,48,49,50]. Therefore, the inhibition of apoptosis through protecting cell proliferation of neurogenesis area is a promising therapeutic strategy in the various brain regions of PD. Indeed, we found that EE induced functional improvement in olfactory and motor functions of PD mice, the increase of cell proliferation, and the decrease apoptotic cells in the brain of PD mice.

Various xenobiotic and environmental toxins are associated with the pathogenesis of PD, including 1-methyl-4-phenyl-1,2,3,6-tetrahydropyridine (MPTP); 6-hydroxydopamine (6-OHDA); pesticides; solvents; metals; and endogenous toxins, including 4-phenylpyridine, *N*-methyl nicotinamide, and *N*-methyl-(R)-salsolinol [51,52,53,54,55,56,57]. As such, the altered capacity for detoxification is a potential risk factor for PD.

Because *CYP* enzymes are responsible for the metabolism of a huge range of endogenous and xenobiotic compounds, they are widely associated not only with the drug metabolism, but also the pathogenesis of various diseases, including metabolic diseases, cardiovascular diseases, malignancies, and neuroinflammatory diseases [58,59,60,61]. Owing to the active function of *CYP* enzymes in neuronal tissues, their roles in various brain disorders are becoming increasingly evident [62]. *CYP1A2* exerts detoxification properties against xenobiotics, in particular MPTP, a well-known toxic material that has been found to induce PD in both animals and humans [63,64,65]. Caffeine consumption has been reported to provide neuroprotection against PD in epidemiological and meta-analysis studies [66,67]. Surprisingly, cigarette smoking is also known to lower the risk of PD [68,69]. It is thought that the induction of *CYP1A2* via caffeine and cigarettes occurs as a mechanism of neuroprotection against the pathogenesis of PD [70,71].

Carbonyl compounds are formed during the peroxidation of lipids and lipoproteins, as well as food poisoning, and are frequently found in various endogenous and xenobiotic compounds. Because reactive carbonyl groups modify proteins and DNA, they are closely associated with various neurodegenerative diseases [72,73,74,75]. Dicarbonyl compounds, which have dual carbonyl groups, are also generated by various oxidative processes, such as oxidative glycation, sugar autoxidation, and lipid peroxidation. The advanced glycation end (AGE) products, derived from modification of proteins by carbonyl and dicarbonyl compounds, induce the release of cytokines, free radicals, and the direct modification of the extracellular matrix and hormonal actions, as well as protein aggregation by oxidative stress. Therefore, AGE products are known to exert a wide range of pathological properties in various diseases, including connective tissue disease, end-stage renal disease, and neurodegenerative diseases [76,77]. *CBR* detoxifies reactive aldehyde, as one of carbonyls derived from lipid peroxidation, and is responsible for neuronal survival and protection against oxidative stress [73]. Because *CRYL1* also catalyzes the detoxification of α-dicarbonyls [77], both enzymes may be associated with the pathogenesis of PD. We think that the extraordinary response of *CBR* in the striatum in our study, which shows significant overexpression in the PD-EE compared to both WT and PD-SC mice, should be elucidated via further experiment.

*PON1* catalyzes the hydrolysis of oxons, which are toxic metabolites of organophosphates [78]. It exerts neuroprotective action in addition to the detoxification of pesticides, including antioxidation, anti-inflammation, and protection against cardiovascular diseases via an inverse correlation with atherosclerosis [78,79,80]. As a result, the mutated *PON1* is known as a risk to PD pathogenesis [80,81,82].

Aldehydes, a type of carbonyl compound, exist ubiquitously in all body tissues. Because they are a very reactive species, various enzymes, such as *CYP*, aldo-ketoreductases, *ADH*, short-chain dehydrogenases/reductases, and *ALDH*, are involved in the metabolism of aldehydes [83]. Aldehydes are catalyzed to the corresponding carboxylic acids via *ALDH* (as a group of nicotinamide adenine dinucleotide phosphate [NAD(P)]+-dependent enzymes). *ADH* and *ALDH* are involved in neuroprotection and antioxidation via the metabolism of vitamin A (retinol) into retinoic acid, the most active form of retinoids. Retinoic acid participates in neuronal differentiation and neural tube patterning via signaling pathways that regulate the gene expression of retinol-binding proteins [84,85]. The dopaminergic system of the central nervous system (CNS) is a target of retinoic acid [86]. The *ALDH1A7* gene is expressed in mice, but not in humans, and shares identical residues of 84% and 91% similarity with *ALDH1A1* in humans and mice, respectively [87]. *ALDH1A1* degrades dopamine-3,4-dihydroxyphenylacetaldehyde (DOPAL) into 3,4-dihydrophenylacetic acid (DOPAC), blocking the α-synuclein-related cytotoxicity of DOPAL [88].

In our preliminary study [22], we chose to examine the olfactory bulb as an induction site of PD. We expanded the interested areas related to other dopaminergic structures, including the brain stem, frontal cortex, and striatum. The activity of detoxifying enzymes was found to be higher in the PD-SC mice and ameliorated by EE in the olfactory bulb and brain stem. On the other hand, these findings were not significantly apparent in the striatum. These results suggest that the activity of detoxifying enzymes vary according to brain region.

It is necessary to consider the characteristics of the olfactory bulb to determine why the results vary among the different regions. The olfactory bulb acts as an inflow route of exogenous compounds due to its direct exposure to the external environment, and directly relays biological information to the CNS [12,89]. The olfactory bulb secretes antioxidants and detoxifying enzymes to provide protection against harmful substances [8].

Vulnerability against oxidative stress differs across brain region. High utilization of oxygen and enriched content of polyunsaturated lipids make the central nervous system more vulnerable to oxidative stress compared to other tissues [73]. Within the brain, the resilience against oxidative stress varies by region [90], with the substantia nigra (SN) being more vulnerable to oxidative stress compared to the other brain regions [91]. Cardoso et al. [92,93] reported differences in the response of the SN and striatum to oxidative stress using sequential studies on rats. Restricting the dietary provision of essential fatty acids, which is crucial for regulating oxidative stress, cell signaling, and apoptosis, research found that lipid peroxidation increased in the SN but not the striatum in young animals (30–42 days old). The activity of superoxide dismutase (SOD) increased in the striatum, while that of catalase decreased in the SN, indicating that the SN was more vulnerable to oxidative stress than the striatum [92]. In a followed subsequent study using older animals (90–110 days old), resilience of the striatum against oxidative stress was found to decrease with age on the basis of the findings of increased lipid peroxidation in both the SN and striatum and decreased SOD activity in the striatum [93]. The striatum is thought to have a different defensive mechanism against dopaminergic cell loss than other regions of the CNS [90]. Therefore, the olfactory bulb has a high burden and dependence on detoxifying enzymes.

In the same course of the disease, and even in the same subject, the disease stages of PD differ among the brain regions. Generally, the olfactory system and lower brain system are affected first. Then, the pathogenesis propagates to other sites, reaching the neocortex last [9]. Thus, we expect that the oxidative stress burden will vary depending on the brain site. In our results, the levels of detoxifying enzymes associated with phase I of the metabolizing process were differently altered by EE in the brain regions of PD mice. These results suggest that the expression pattern for effect of EE on the enzymes is differently altered depending on the brain regions.

The significance of this research is that EE can regulate to the activity of enzymes participating in the detoxification metabolism in terms of neuroprotection. Normalized levels of detoxifying enzymes as a result of EE suggest that voluntary exercise can reduce the metabolic burden of detoxification, including neuroprotection.

In this study, we provided evidence that EE has neurobehavioral improvement effects, reducing the GSH/GSSG ratio that is decreased when the cells are in oxidative stress. We also showed neuroprotective effects such as apoptosis and the protection of cell proliferation, as well as the alternation of detoxifying enzymes by EE in the brain. Taken together, EE reduces oxidative stress and apoptosis, and induces the protection of cell proliferation, eventually normalizing the expression of detoxifying enzymes which metabolize toxic molecules.

## 5. Conclusions

In a PD mouse model, the activity of detoxifying enzymes increased in the olfactory bulb and brain stem during the early stage of disease, and this event was stabilized by EE. However, in the other dopaminergic structures, namely, the striatum, the enzymatic activity was not consistent to the responses in the olfactory bulb and brain stem. Thus, the strategy for treating the pathogenesis of PD may vary from region to region in the brain. Enzymatic activity and motor functions, as well as olfactory function, were improved by EE. Our results indicate that rehabilitation has a protective effect against neuronal deterioration in PD and subsequently decreases detoxifying burden.

## Figures and Tables

**Table 1:** List of primers used for qRT-PCR.

Gene ID	Genes	Primer Sequences	
13077	*Cyp1a2*	Forward	GCTTCTCCATAGCCTCGGAC
		Reverse	TTAGCCACCGATTCCACCAC
12409	*Cbr2*	Forward	CCCCCTTCCACATTCAAGCA
		Reverse	CTCCTCTGTGATGGTCTCGC
68631	*Cryl1*	Forward	GATTGACGGCTTCGTCCTGA
		Reverse	GCATAGTCTCCAAGGGTCCG
18979	*Pon1*	Forward	ATGACGCAGAGAATCCTCCC
		Reverse	TTTGTACACAGAGGCGACCG
11522	*Adh1*	Forward	GACATAGAAGTCGCACCCCC
		Reverse	CCAACGCTCTCAACAATGCC
26358	*Aldh1a7*	Forward	GGTTTAGCAGCAGGAGTCTTCA
		Reverse	CAGCCAAATAGCAGTTCACCC
14433	*Gapdh*	Forward	CAAGGTCATCCATGACAACTTTG
		Reverse	GTCCACCACCCTGTTGCTGTAG

*Cyp1a2*, cytochrome P450 family 1 subfamily A member 2; *Cbr2*, carbonyl reductase 2; *Cryl1*, crystallin lambda 1; *Pon1*, paraoxonase 1; *Adh1*, alcohol dehydrogenase 1; *Aldh1a7*, aldehyde dehydrogenase 1A7; *Gapdh*, glyceraldehyde-3-phosphate dehydrogenase.

## References

[1] Jagmag S.A., Tripathi N., Shukla S.D., Maiti S., Khurana S. (2015). Evaluation of Models of Parkinson’s Disease. Front. Neurosci..

[2] Seo W.K., Koh S.B., Kim B.J., Yu S.W., Park M.H., Park K.W., Lee D.H. (2007). Prevalence of Parkinson’s disease in Korea. J. Clin. Neurosci..

[3] Balestrino R., Schapira A.H.V. (2020). Parkinson disease. Eur. J. Neurol..

[4] Reichmann H., Jost W. (2008). Parkinson’s disease—Many diseases with many faces. J. Neurol..

[5] Procaccini C., Santopaolo M., Faicchia D., Colamatteo A., Formisano L., de Candia P., Galgani M., De Rosa V., Matarese G. (2016). Role of metabolism in neurodegenerative disorders. Metabolism.

[6] Nebert D.W., Dalton T.P. (2006). The role of cytochrome P450 enzymes in endogenous signalling pathways and environmental carcinogenesis. Nat. Rev. Cancer.

[7] Ravindranath V., Strobel H.W. (2013). Cytochrome P450-mediated metabolism in brain: Functional roles and their implications. Expert Opin. Drug Metab. Toxicol..

[8] Heydel J.M., Coelho A., Thiebaud N., Legendre A., Le Bon A.M., Faure P., Neiers F., Artur Y., Golebiowski J., Briand L. (2013). Odorant-binding proteins and xenobiotic metabolizing enzymes: Implications in olfactory perireceptor events. Anat. Rec..

[9] Braak H., Rub U., Gai W.P., Del Tredici K. (2003). Idiopathic Parkinson’s disease: Possible routes by which vulnerable neuronal types may be subject to neuroinvasion by an unknown pathogen. J. Neural Transm..

[10] Del Tredici K., Rub U., De Vos R.A., Bohl J.R., Braak H. (2002). Where does parkinson disease pathology begin in the brain?. J. Neuropathol. Exp. Neurol..

[11] Lionnet A., Leclair-Visonneau L., Neunlist M., Murayama S., Takao M., Adler C.H., Derkinderen P., Beach T.G. (2018). Does Parkinson’s disease start in the gut?. Acta Neuropathol..

[12] Minn A., Leclerc S., Heydel J.M., Minn A.L., Denizcot C., Cattarelli M., Netter P., Gradinaru D. (2002). Drug transport into the mammalian brain: The nasal pathway and its specific metabolic barrier. J. Drug Target..

[13] Seo J.H., Pyo S., Shin Y.K., Nam B.G., Kang J.W., Kim K.P., Lee H.Y., Cho S.R. (2018). The Effect of Environmental Enrichment on Glutathione-Mediated Xenobiotic Metabolism and Antioxidation in Normal Adult Mice. Front. Neurol..

[14] Rey N.L., Wesson D.W., Brundin P. (2018). The olfactory bulb as the entry site for prion-like propagation in neurodegenerative diseases. Neurobiol. Dis..

[15] Kalia L.V., Lang A.E. (2015). Parkinson’s disease. Lancet.

[16] Lee M.Y., Yu J.H., Kim J.Y., Seo J.H., Park E.S., Kim C.H., Kim H., Cho S.R. (2013). Alteration of synaptic activity-regulating genes underlying functional improvement by long-term exposure to an enriched environment in the adult brain. Neurorehabilit. Neural Repair.

[17] Will B., Galani R., Kelche C., Rosenzweig M.R. (2004). Recovery from brain injury in animals: Relative efficacy of environmental enrichment, physical exercise or formal training (1990–2002). Prog. Neurobiol..

[18] Seo J.H., Yu J.H., Suh H., Kim M.S., Cho S.R. (2013). Fibroblast growth factor-2 induced by enriched environment enhances angiogenesis and motor function in chronic hypoxic-ischemic brain injury. PLoS ONE.

[19] Mohammed A.H., Zhu S.W., Darmopil S., Hjerling-Leffler J., Ernfors P., Winblad B., Diamond M.C., Eriksson P.S., Bogdanovic N. (2002). Environmental enrichment and the brain. Prog. Brain Res..

[20] Kempermann G., Gast D., Gage F.H. (2002). Neuroplasticity in old age: Sustained fivefold induction of hippocampal neurogenesis by long-term environmental enrichment. Ann. Neurol..

[21] Ravikiran T., Sowbhagya R., Anupama S.K., Anand S., Bhagyalakshmi D. (2016). Age-related changes in the brain antioxidant status: Modulation by dietary supplementation of Decalepis hamiltonii and physical exercise. Mol. Cell. Biochem..

[22] Wi S., Lee J.W., Kim M., Park C.H., Cho S.R. (2018). An Enriched Environment Ameliorates Oxidative Stress and Olfactory Dysfunction in Parkinson’s Disease with alpha-Synucleinopathy. Cell Transplant..

[23] Smith T.W., Lippa C.F. (1995). Ki-67 immunoreactivity in Alzheimer’s disease and other neurodegenerative disorders. J. Neuropathol. Exp. Neurol..

[24] Alvarez-Buylla A., Garcia-Verdugo J.M. (2002). Neurogenesis in adult subventricular zone. J. Neurosci..

[25] Farrell K.F., Krishnamachari S., Villanueva E., Lou H., Alerte T.N., Peet E., Drolet R.E., Perez R.G. (2014). Non-motor parkinsonian pathology in aging A53T α-synuclein mice is associated with progressive synucleinopathy and altered enzymatic function. J. Neurochem..

[26] Fan L.W., Lin S., Pang Y., Lei M., Zhang F., Rhodes P.G., Cai Z. (2005). Hypoxia-ischemia induced neurological dysfunction and brain injury in the neonatal rat. Behav. Brain Res..

[27] Tomlinson C.L., Herd C.P., Clarke C.E., Meek C., Patel S., Stowe R., Deane K.H., Shah L., Sackley C.M., Wheatley K. (2014). Physiotherapy for Parkinson’s disease: A comparison of techniques. Cochrane Database Syst. Rev..

[28] Palasz E., Niewiadomski W., Gasiorowska A., Wysocka A., Stepniewska A., Niewiadomska G. (2019). Exercise-Induced Neuroprotection and Recovery of Motor Function in Animal Models of Parkinson’s Disease. Front. Neurol..

[29] Van der Kolk N.M., King L.A. (2013). Effects of exercise on mobility in people with Parkinson’s disease. Mov. Disord..

[30] Stuckenschneider T., Askew C.D., Meneses A.L., Baake R., Weber J., Schneider S. (2019). The Effect of Different Exercise Modes on Domain-Specific Cognitive Function in Patients Suffering from Parkinson’s Disease: A Systematic Review of Randomized Controlled Trials. J. Parkinsons Dis..

[31] Alves Da Rocha P., McClelland J., Morris M.E. (2015). Complementary physical therapies for movement disorders in Parkinson’s disease: A systematic review. Eur. J. Phys. Rehabil. Med..

[32] Monteiro-Junior R.S., Cevada T., Oliveira B.R., Lattari E., Portugal E.M., Carvalho A., Deslandes A.C. (2015). We need to move more: Neurobiological hypotheses of physical exercise as a treatment for Parkinson’s disease. Med. Hypotheses.

[33] Sehm B., Taubert M., Conde V., Weise D., Classen J., Dukart J., Draganski B., Villringer A., Ragert P. (2014). Structural brain plasticity in Parkinson’s disease induced by balance training. Neurobiol. Aging.

[34] Fisher B.E., Li Q., Nacca A., Salem G.J., Song J., Yip J., Hui J.S., Jakowec M.W., Petzinger G.M. (2013). Treadmill exercise elevates striatal dopamine D2 receptor binding potential in patients with early Parkinson’s disease. Neuroreport.

[35] Beall E.B., Lowe M.J., Alberts J.L., Frankemolle A.M., Thota A.K., Shah C., Phillips M.D. (2013). The effect of forced-exercise therapy for Parkinson’s disease on motor cortex functional connectivity. Brain Connect..

[36] Komitova M., Mattsson B., Johansson B.B., Eriksson P.S. (2005). Enriched environment increases neural stem/progenitor cell proliferation and neurogenesis in the subventricular zone of stroke-lesioned adult rats. Stroke.

[37] Veena J., Srikumar B.N., Mahati K., Bhagya V., Raju T.R., Shankaranarayana Rao B.S. (2009). Enriched environment restores hippocampal cell proliferation and ameliorates cognitive deficits in chronically stressed rats. J. Neurosci. Res..

[38] Okuda H., Tatsumi K., Makinodan M., Yamauchi T., Kishimoto T., Wanaka A. (2009). Environmental enrichment stimulates progenitor cell proliferation in the amygdala. J. Neurosci. Res..

[39] Young D., Lawlor P.A., Leone P., Dragunow M., During M.J. (1999). Environmental enrichment inhibits spontaneous apoptosis, prevents seizures and is neuroprotective. Nat. Med..

[40] Chen X., Zhang X., Xue L., Hao C., Liao W., Wan Q. (2017). Treatment with Enriched Environment Reduces Neuronal Apoptosis in the Periinfarct Cortex after Cerebral Ischemia/Reperfusion Injury. Cell. Physiol. Biochem..

[41] Hoglinger G.U., Rizk P., Muriel M.P., Duyckaerts C., Oertel W.H., Caille I., Hirsch E.C. (2004). Dopamine depletion impairs precursor cell proliferation in Parkinson disease. Nat. Neurosci..

[42] Singh S., Dikshit M. (2007). Apoptotic neuronal death in Parkinson’s disease: Involvement of nitric oxide. Brain Res. Rev..

[43] Andersen J.K. (2001). Does neuronal loss in Parkinson’s disease involve programmed cell death?. Bioessays.

[44] Tatton W.G., Chalmers-Redman R., Brown D., Tatton N. (2003). Apoptosis in Parkinson’s disease: Signals for neuronal degradation. Ann. Neurol..

[45] Ekshyyan O., Aw T.Y. (2004). Apoptosis: A key in neurodegenerative disorders. Curr. Neurovasc. Res..

[46] Jungling A., Reglodi D., Karadi Z.N., Horvath G., Farkas J., Gaszner B., Tamas A. (2017). Effects of Postnatal Enriched Environment in a Model of Parkinson’s Disease in Adult Rats. Int. J. Mol. Sci..

[47] Steiner B., Winter C., Hosman K., Siebert E., Kempermann G., Petrus D.S., Kupsch A. (2006). Enriched environment induces cellular plasticity in the adult substantia nigra and improves motor behavior function in the 6-OHDA rat model of Parkinson’s disease. Exp. Neurol..

[48] Nithianantharajah J., Hannan A.J. (2006). Enriched environments, experience-dependent plasticity and disorders of the nervous system. Nat. Rev. Neurosci..

[49] Sha J.C., Ismail R., Marlena D., Lee J.L. (2016). Environmental complexity and feeding enrichment can mitigate effects of space constraints in captive callitrichids. Lab. Anim..

[50] Tsai P.P., Stelzer H.D., Hedrich H.J., Hackbarth H. (2003). Are the effects of different enrichment designs on the physiology and behaviour of DBA/2 mice consistent?. Lab. Anim..

[51] Yan D., Zhang Y., Liu L., Shi N., Yan H. (2018). Pesticide exposure and risk of Parkinson’s disease: Dose-response meta-analysis of observational studies. Regul. Toxicol. Pharmacol..

[52] Langston J.W. (2017). The MPTP Story. J. Parkinsons Dis..

[53] Naoi M., Maruyama W., Akao Y., Yi H. (2002). Dopamine-derived endogenous N-methyl-(R)-salsolinol: Its role in Parkinson’s disease. Neurotoxicol. Teratol..

[54] Williams A.C., Cartwright L.S., Ramsden D.B. (2005). Parkinson’s disease: The first common neurological disease due to auto-intoxication?. QJM.

[55] Hamadjida A., Frouni I., Kwan C., Huot P. (2019). Classic animal models of Parkinson’s disease: A historical perspective. Behav. Pharmacol..

[56] Tanner C.M., Kamel F., Ross G.W., Hoppin J.A., Goldman S.M., Korell M., Marras C., Bhudhikanok G.S., Kasten M., Chade A.R. (2011). Rotenone, paraquat, and Parkinson’s disease. Environ. Health Perspect..

[57] Goldman S.M. (2014). Environmental toxins and Parkinson’s disease. Annu. Rev. Pharmacol. Toxicol..

[58] Dos Santos L.R.B., Fleming I. (2019). Role of cytochrome P450-derived, polyunsaturated fatty acid mediators in diabetes and the metabolic syndrome. Prostaglandins Other Lipid Mediat..

[59] Manikandan P., Nagini S. (2018). Cytochrome P450 Structure, Function and Clinical Significance: A Review. Curr. Drug Targets.

[60] Elfaki I., Mir R., Almutairi F.M., Duhier F.M.A. (2018). Cytochrome P450: Polymorphisms and Roles in Cancer, Diabetes and Atherosclerosis. Asian Pac. J. Cancer Prev..

[61] Fatunde O.A., Brown S.A. (2020). The Role of CYP450 Drug Metabolism in Precision Cardio-Oncology. Int. J. Mol. Sci..

[62] Ghosh C., Hossain M., Solanki J., Dadas A., Marchi N., Janigro D. (2016). Pathophysiological implications of neurovascular P450 in brain disorders. Drug Discov. Today.

[63] Sonsalla P.K., Heikkila R.E. (1986). The influence of dose and dosing interval on MPTP-induced dopaminergic neurotoxicity in mice. Eur. J. Pharmacol..

[64] Apte S.N., Langston J.W. (1983). Permanent neurological deficits due to lithium toxicity. Ann. Neurol..

[65] Kopin I.J., Markey S.P. (1988). MPTP toxicity: Implications for research in Parkinson’s disease. Annu. Rev. Neurosci..

[66] Lim J.L., Wilhelmus M.M., de Vries H.E., Drukarch B., Hoozemans J.J., van Horssen J. (2014). Antioxidative defense mechanisms controlled by Nrf2: State-of-the-art and clinical perspectives in neurodegenerative diseases. Arch. Toxicol..

[67] Kolahdouzan M., Hamadeh M.J. (2017). The neuroprotective effects of caffeine in neurodegenerative diseases. CNS Neurosci. Ther..

[68] Ritz B., Ascherio A., Checkoway H., Marder K.S., Nelson L.M., Rocca W.A., Ross G.W., Strickland D., Van Den Eeden S.K., Gorell J. (2007). Pooled analysis of tobacco use and risk of Parkinson disease. Arch. Neurol..

[69] Breckenridge C.B., Berry C., Chang E.T., Sielken R.L., Mandel J.S. (2016). Association between Parkinson’s Disease and Cigarette Smoking, Rural Living, Well-Water Consumption, Farming and Pesticide Use: Systematic Review and Meta-Analysis. PLoS ONE.

[70] Das N.P., Shahi G.S., Moochhala S.M., Sato T., Sunamoto J. (1992). Effect of 1-methyl-4-phenyl-1,2,3,6-tetrahydropyridine (MPTP) and its toxic metabolites on the physicochemical property of the liposomal membrane in relation to their cytochrome P-450 inhibition. Chem. Phys. Lipids.

[71] Singh S., Singh K., Gupta S.P., Patel D.K., Singh V.K., Singh R.K., Singh M.P. (2009). Effect of caffeine on the expression of cytochrome P450 1A2, adenosine A2A receptor and dopamine transporter in control and 1-methyl 4-phenyl 1, 2, 3, 6-tetrahydropyridine treated mouse striatum. Brain Res..

[72] Oppermann U. (2007). Carbonyl reductases: The complex relationships of mammalian carbonyl- and quinone-reducing enzymes and their role in physiology. Annu. Rev. Pharmacol. Toxicol..

[73] Maser E. (2006). Neuroprotective role for carbonyl reductase?. Biochem. Biophys. Res. Commun..

[74] Dalle-Donne I., Giustarini D., Colombo R., Rossi R., Milzani A. (2003). Protein carbonylation in human diseases. Trends Mol. Med..

[75] Curtis J.M., Hahn W.S., Long E.K., Burrill J.S., Arriaga E.A., Bernlohr D.A. (2012). Protein carbonylation and metabolic control systems. Trends Endocrinol. Metab..

[76] Ajith T.A., Vinodkumar P. (2016). Advanced Glycation End Products: Association with the Pathogenesis of Diseases and the Current Therapeutic Advances. Curr. Clin. Pharmacol..

[77] Ishikura S., Usami N., Araki M., Hara A. (2005). Structural and functional characterization of rabbit and human L-gulonate 3-dehydrogenase. J. Biochem..

[78] Androutsopoulos V.P., Kanavouras K., Tsatsakis A.M. (2011). Role of paraoxonase 1 (PON1) in organophosphate metabolism: Implications in neurodegenerative diseases. Toxicol. Appl. Pharmacol..

[79] Furlong C.E., Suzuki S.M., Stevens R.C., Marsillach J., Richter R.J., Jarvik G.P., Checkoway H., Samii A., Costa L.G., Griffith A. (2010). Human PON1, a biomarker of risk of disease and exposure. Chem. Biol. Interact..

[80] Menini T., Gugliucci A. (2014). Paraoxonase 1 in neurological disorders. Redox Rep..

[81] Clarimon J., Eerola J., Hellström O., Tienari P.J., Singleton A. (2004). Paraoxonase 1 (PON1) gene polymorphisms and Parkinson’s disease in a Finnish population. Neurosci. Lett..

[82] Akhmedova S.N., Yakimovsky A.K., Schwartz E.I. (2001). Paraoxonase 1 Met--Leu 54 polymorphism is associated with Parkinson’s disease. J. Neurol. Sci..

[83] Ahmed Laskar A., Younus H. (2019). Aldehyde toxicity and metabolism: The role of aldehyde dehydrogenases in detoxification, drug resistance and carcinogenesis. Drug Metab. Rev..

[84] Tafti M., Ghyselinck N.B. (2007). Functional implication of the vitamin A signaling pathway in the brain. Arch. Neurol..

[85] MacDonald P.N., Bok D., Ong D.E. (1990). Localization of cellular retinol-binding protein and retinol-binding protein in cells comprising the blood-brain barrier of rat and human. Proc. Natl. Acad. Sci. USA.

[86] Takeda A., Nyssen O.P., Syed A., Jansen E., Bueno-de-Mesquita B., Gallo V. (2014). Vitamin A and carotenoids and the risk of Parkinson’s disease: A systematic review and meta-analysis. Neuroepidemiology.

[87] Cai H., Liu G., Sun L., Ding J. (2014). Aldehyde Dehydrogenase 1 making molecular inroads into the differential vulnerability of nigrostriatal dopaminergic neuron subtypes in Parkinson’s disease. Transl. Neurodegener..

[88] Marchitti S.A., Deitrich R.A., Vasiliou V. (2007). Neurotoxicity and metabolism of the catecholamine-derived 3,4-dihydroxyphenylacetaldehyde and 3,4-dihydroxyphenylglycolaldehyde: The role of aldehyde dehydrogenase. Pharmacol. Rev..

[89] Zhang Q., Yan W., Bai Y., Zhu Y., Ma J. (2014). Repeated formaldehyde inhalation impaired olfactory function and changed SNAP25 proteins in olfactory bulb. Int. J. Occup. Environ. Health.

[90] Jensen N., Oliveira J.R. (2014). Basal ganglia vulnerability to oxidative stress. Front. Neurosci..

[91] Kidd P.M. (2000). Parkinson’s disease as multifactorial oxidative neurodegeneration: Implications for integrative management. Altern. Med. Rev..

[92] Cardoso H.D., Passos P.P., Lagranha C.J., Ferraz A.C., Santos Junior E.F., Oliveira R.S., Oliveira P.E., Santos Rde C., Santana D.F., Borba J.M. (2012). Differential vulnerability of substantia nigra and corpus striatum to oxidative insult induced by reduced dietary levels of essential fatty acids. Front. Hum. Neurosci..

[93] Cardoso H.D., dos Santos Junior E.F., de Santana D.F., Goncalves-Pimentel C., Angelim M.K., Isaac A.R., Lagranha C.J., Guedes R.C., Beltrao E.I., Morya E. (2014). Omega-3 deficiency and neurodegeneration in the substantia nigra: Involvement of increased nitric oxide production and reduced BDNF expression. Biochim. Biophys. Acta.

